# Spatial Patterns and Socioecological Drivers of Dengue Fever Transmission in Queensland, Australia

**DOI:** 10.1289/ehp.1003270

**Published:** 2011-10-20

**Authors:** Wenbiao Hu, Archie Clements, Gail Williams, Shilu Tong, Kerrie Mengersen

**Affiliations:** 1 School of Population Health, The University of Queensland, Brisbane, Queensland, Australia; 2 School of Public Health, and; 3 School of Mathematical Sciences, Queensland University of Technology, Brisbane, Queensland, Australia

**Keywords:** Bayesian spatial analysis, dengue, socioecological factors

## Abstract

Background: Understanding how socioecological factors affect the transmission of dengue fever (DF) may help to develop an early warning system of DF.

Objectives: We examined the impact of socioecological factors on the transmission of DF and assessed potential predictors of locally acquired and overseas-acquired cases of DF in Queensland, Australia.

Methods: We obtained data from Queensland Health on the numbers of notified DF cases by local government area (LGA) in Queensland for the period 1 January 2002 through 31 December 2005. Data on weather and the socioeconomic index were obtained from the Australian Bureau of Meteorology and the Australian Bureau of Statistics, respectively. A Bayesian spatial conditional autoregressive model was fitted at the LGA level to quantify the relationship between DF and socioecological factors.

Results: Our estimates suggest an increase in locally acquired DF of 6% [95% credible interval (CI): 2%, 11%] and 61% (95% CI: 2%, 241%) in association with a 1-mm increase in average monthly rainfall and a 1°C increase in average monthly maximum temperature between 2002 and 2005, respectively. By contrast, overseas-acquired DF cases increased by 1% (95% CI: 0%, 3%) and by 1% (95% CI: 0%, 2%) in association with a 1-mm increase in average monthly rainfall and a 1-unit increase in average socioeconomic index, respectively.

Conclusions: Socioecological factors appear to influence the transmission of DF in Queensland, but the drivers of locally acquired and overseas-acquired DF may differ. DF risk is spatially clustered with different patterns for locally acquired and overseas-acquired cases.

Dengue fever (DF) is one of the most prevalent arboviral diseases in the world, and its global range of transmission has increased significantly in recent decades (Phillips 2008). Secondary DF infection with a serotype of dengue virus different from that of the primary infection commonly results in the more serious dengue hemorrhagic fever (Gubler 1998). The large-scale reemergence of DF during the past few decades has renewed the status of DF as a serious international public health problem, especially in tropical and subtropical areas, including Australia (Gubler 1998; Rogers et al. 2006). Over the past 17 years (1993–2009), 6,271 laboratory-confirmed DF cases have been reported to the Australian Department of Health and Ageing (2010). Major outbreaks have occurred in northern Queensland, centered in Cairns, Townsville, and the Torres Strait islands (Hanna et al. 1998, 2001; Tropical Public Health Unit Network 2004). Although DF is not naturally endemic in Australia, the dengue vector—*Aedes aegypti*—inhabits northern Queensland, and outbreaks can occur when the virus is introduced to the local mosquito population by infected international travelers and migrants or residents who were infected while traveling overseas (Tropical Public Health Unit Network 2004). The recent arrival of the exotic species—*Aedes albopictus*—into Australia is of greater concern for southern Australia (Russell 2009). If *Ae. albopictus* becomes colonized on the mainland, it could very likely extend to all the southern states (Russell et al. 2005), broadening the potential geographical range of dengue transmission in Australia. Currently, no antidengue drugs are available, and no effective vaccine is available for DF (Edelman 2005).

Weather conditions directly affect the breeding, survival, and abundance of mosquitoes (Hales et al. 2002). The ideal temperature range for transmission of DF is 18–33.2°C, with female mosquitoes feeding more frequently when temperatures are higher (Depradine and Lovell 2004; Nagao et al. 2003). Some studies show that meteorological variables (e.g., rainfall, temperature, and relative humidity) are important climatic factors that could influence the risk of DF outbreaks (Depradine and Lovell 2004; Diallo et al. 2003). These variables can be modeled to predict the onset and severity of DF epidemics. Social and economic factors may also contribute to DF transmission (Mondini and Chiaraualloti-neto 2008). Traveling to DF-endemic regions may also increase the risk of transmission (Schwartz et al. 2008; Wilder-Smith and Gubler 2008; Wilder-Smith and Schwartz 2005). Unplanned urbanization and declining and inadequate public health resources for vector control are also key factors that promote dengue transmission (Gubler and Clark 1996). However, existing forecasting models for DF usually consider one set of variables (e.g., climate variability) and do not account for socioecological factors such as travel, sociodemographic characteristics, or interactions between climate variables and socioeconomic factors.

In Australia, current DF surveillance focuses on detecting imported cases, because a viremic traveler (imported case) could readily initiate an outbreak. However, an outbreak is declared only after a locally acquired case becomes confirmed (Tropical Public Health Unit Network 2004). Therefore, assessing both imported and locally acquired cases is crucial for modeling DF epidemic dynamics and evaluating the risk of DF (Degallier et al. 2009).

Bayesian spatial models provide a flexible and rigorous approach for multilevel spatial analysis and disease mapping (Best et al. 2005). They are increasingly being used to estimate spatial variation in infectious disease risk among spatially aggregated units and associated uncertainty (Hu et al. 2010c). These models can offer suitable platforms for incorporating and estimating spatial correlation while simultaneously estimating covariate effects.

Our previous study showed that there has been an increase in DF cases in southeast Queensland (Hu et al. 2010b). However, the underlying causes of changes in spatial patterns of DF need further investigation. In the present study, we examined the potential impact of socioecological factors on DF in Queensland and assessed differences in spatial patterns and predictors of locally acquired and overseas-acquired DF in Queensland, Australia.

## Materials and Methods

*Study area.* Queensland is located in northeast Australia between latitudes 10–28° S and longitudes 138–153° E; it covers around 1,727,200 km^2^, with 7,400 km of mainland coastline. There is significant variation in climate across the state. Low rainfall and hot summers occur in the inland west. A monsoonal pattern of wet and dry seasons is typical for the far north, and warm temperate conditions occur along the coastal strip. Queensland has an average temperature of 25°C in summer and 15°C in winter. The average annual rainfall of about 1,000 mm falls mostly between January and March and ranges from < 150 mm/year in the southwest to > 4,000 mm/year on the far northern coast. There are 125 local government areas (LGAs) in Queensland, with populations ranging from 312 to 888,449 people (Australian Bureau of Statistics 2010).

*Data collection.* We obtained data from Queensland Health (the state government department of health, Brisbane, Australia) on numbers of notified DF cases by LGA, acquired both locally and overseas, for the period 1 January 2002 to 31 December 2005. Because DF is a notifiable infectious disease, it is a legal requirement for laboratories to report positive test results to the Communicable Disease Unit at Queensland Health, where the data are archived. These DF notification data were entered into a digital base map of LGAs using a geographic information system (GIS). We also obtained data on weather (including temperature and rainfall) from the Australian Bureau of Meteorology (Brisbane, Australia), including interpolated monthly mean maximum temperature and monthly precipitation between January 2002 and December 2005, which were available at a 0.25° × 0.25° grid resolution. Average temperature and rainfall values for each LGA during the study period were extracted using the GIS software package Vertical Map, version 3.0 (MapInfo Corporation 2003). We obtained data for the same time period for each LGA on the socioeconomic index for areas (SEIFA) and sociodemographic factors, including population size and numbers of overseas travelers, from the Australian Bureau of Statistics (2010). SEIFA index values are derived from multiple-weighted variables that take into account variables relating to education, occupation, wealth and living conditions, and so forth. SEIFA can provide a rank of the level of social and economic well-being by regions; lower values indicate lower socioeconomic status. We modeled SEIFA as a continuous variable because its distribution was not highly skewed, and we did not want to lose information or reduce statistical power by categorizing the variable.

*Data analysis.* Separate Poisson regression models were developed in a Bayesian framework for locally acquired and overseas-acquired cases, using WinBUGS software, version 1.4.3 (MRC Biostatistics Unit 2008). These models assumed that the observed counts of DF cases (*O_k_*) for the *k*th LGA (*k* = 1 . . . 125) followed a Poisson distribution with mean μ*_k_*:

*O_k_* ~ Poisson(μ*_k_*) [1]

and

log(μ*_k_*) = log(*E_k_*) + θ*_k_*, [2]

where *E_k_* (the expected number of cases in LGA *k*) is an offset to control for population size. The mean log relative risk (RR), θ*_k_*, for each predictor was modeled as

θ*_k_* = α + (Temp*_k_*)β_1_ + (Rain*_k_*)β_2_ + (SEIFA*_k_*)β_3_ + (Visitors*_k_*)β_4_ + *u_k_* + *v_k_*, [3]

where α is the intercept, β_1_ is the coefficient for temperature, β_2_ is the coefficient for rainfall, β_3_ is the coefficient for SEIFA, β_4_ is the proportion of overseas visitors in LGA *k* (numbers of overseas visitors and population of LGA), *u_k_* is a spatially structured random effect with mean zero and variance σ*_u_*^2^, and *v_k_* is a spatially unstructured random effect with mean zero and variance σ*_v_*^2^. Modeled values for temperature, rainfall, and SEIFA were centered around the mean values for each variable. Spatial structuring in *u_k_* was modeled using a conditional autoregressive (CAR) prior structure, with spatial relationships between LGAs modeled using a simple adjacency weights matrix (Lawson et al. 2003). Thus, *u_k_* has a normal distribution with conditional weighted mean given by the simple average of the neighbors of *u_k_* and conditional variance inversely proportional to the number of neighbors.

We conducted an initial burn-in of 5,000 iterations that were subsequently discarded. Convergence was assessed by visual inspection of posterior density plots, history plots, and autocorrelation of selected parameters. Convergence was reached within the first 20,000 iterations for the model. A subsequent set of 40,000 iterations was used for more accurate estimation (MRC Biostatistics Unit 2003). Model selection was undertaken using the deviance information criterion (DIC), where a lower DIC indicates a better trade-off between model fit and parsimony. In addition, models of locally acquired DF were run with and without data for a single outlier LGA (defined as observations well outside the main body of the data). In Poisson regression models, main effects and interaction effects were both considered. In all analyses, we adopted an α-level of 0.05 to indicate statistical significance.

## Results

Table 1 shows the summary statistics for each variable. The mean monthly numbers of locally acquired and overseas-acquired DF cases were 7.89 and 1.54, respectively; the mean monthly maximum temperature, rainfall, SEIFA, and proportion of overseas visitors were 28.55°C, 57.81 mm, 935.61, and 12.27‰ (per thousand), respectively.

**Table 1:** Descriptive statistics of DF and socioecological factors by LGA in Queensland, January 2002 through December 2005.

Variable	Mean ± SD	Range
DF*a*				
Locally acquired		7.89 ± 54.81		0–521
Overseas acquired		1.54 ± 7.31		0–76
Temperature (°C)*b*		28.55 ± 2.47		23.96–34.11
Rainfall (mm)*c*		57.80 ± 30.43		10.61–260.44
SEIFA		935.61 ± 41.63		831.36–1059.84
Proportion of overseas visitors (‰)*d*		12.27 ± 24.49		0–219.23
**a**Numbers of locally acquired DF by LGA and of overseas-acquired DF. **b**Average maximum temperature by LGA. **c**Average rainfall by LGA. **d**Number of overseas visitors and population of LGA (per thousand).				

**Figure 1:**
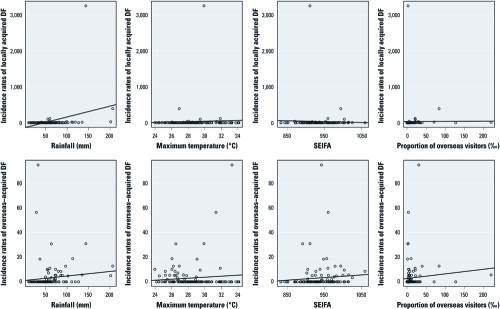
Scatterplot with regression lines of DF incidence rates and explanatory variables.

Scatterplots with regression lines in Figure 1 depict the crude relationships between the dependent and independent variables. These plots reveal that incidence rates of locally acquired DF were positively associated with average rainfall and maximum temperature, whereas incidence rates of overseas-acquired DF were positively associated with all the independent variables. One LGA had an extremely large number of locally acquired DF cases (512 cases, compared with the next highest value of 316 cases).

**Figure 2:**
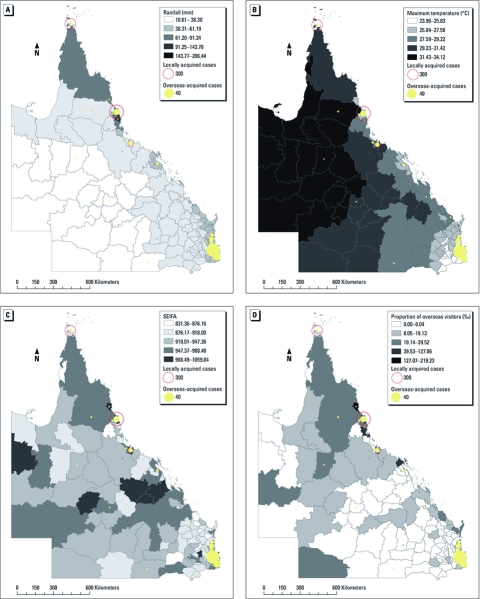
Locally acquired DF and overseas-acquired DF by rainfall (*A*), maximum temperature (*B*), SEIFA (*C*), and proportion of overseas travel (*D*), by LGA in Queensland.

Figure 2 shows the spatial patterns of average monthly rainfall, maximum temperature, proportion of overseas visitors, and SEIFA in Queensland by LGA, as well as numbers of locally acquired and overseas-acquired cases. These maps confirm variation in these characteristics by geographical location.

The estimated increase in locally acquired DF cases was 6% [95% credible interval (CI): 2%, 11%] and 61% (95% CI: 2%, 241%) for a 1-mm increase in average monthly rainfall and a 1°C increase in average monthly maximum temperature, respectively (Table 2). The expected increase in overseas-acquired DF cases was 1% (95% CI: 0%, 3%) and 1% (95% CI: 0%, 2%) for a 1-mm increase in average rainfall and a 1-unit increase in SEIFA score, respectively. No substantial associations were observed between locally acquired DF and the proportion of overseas visitors or SEIFA, or between overseas-acquired DF and maximum temperature or the proportion of overseas visitors. The estimated spatial variation (*u*; mean ± SD = 0.745 ± 0.745 for locally acquired cases and 0.331 ± 0.363 for overseas-acquired cases) was small relative to the remaining unstructured variation (*v*; 2.346 ± 0.707 for locally acquired cases and 0.483 ± 0.258 for overseas-acquired cases; Table 2). The spatial variation is the variation that is spatially structured (i.e., demonstrates spatial autocorrelation), after accounting for the model covariates (which themselves may explain some of the spatial structure of the data). The unstructured variation is the component that is spatially random. We tested interactions between rainfall, temperature, SEIFA, and proportion of overseas visitors in our models. However, no significant interactions were found among the variables (*p* > 0.05). Spatial models that included both spatially structured and unstructured random effects had the smallest DIC (136 and 183 for models of locally acquired and overseas-acquired DF, respectively, vs. 138 and 185 for models with a spatially structured random effect only and 1,646 and 198 for models without random effects).

**Table 2:** Regression coefficients from Bayesian spatial CAR models of DF in Queensland, Australia.

Variable	Posterior mean ± SD	Monte Carlo error	RR (95%CI)
Model 1: locally acquired cases						
Intercept		–5.349 ± 1.004		0.04		
Rainfall (mm)		0.061 ± 0.019		< 0.01		1.06 (1.02, 1.11)
Temperature (°C)		0.476 ± 0.213		< 0.01		1.61 (1.03, 2.41)
SEIFA		0.004 ± 0.012		< 0.01		1.00 (0.98, 1.03)
Proportion of overseas visitors (‰)		–0.002 ± 0.016		< 0.01		0.99 (0.97, 1.03)
Heterogeneity						
Structured (*u*)		0.745 ± 0.745		0.08		
Unstructured (*v*)		2.346 ± 0.707		0.04		
Model 2: overseas-acquired cases						
Intercept		–0.973 ± 0.2763		< 0.01		
Rainfall (mm)		0.014 ± 0.005		< 0.01		1.01 (1.00, 1.03)
Temperature (°C)		–0.048 ± 0.004		< 0.01		0.95 (0.77, 1.14)
SEIFA		0.008 ± 0.004		< 0.01		1.01 (1.00, 1.02)
Proportion of overseas visitors (‰)		–0.002 ± 0.016		< 0.01		0.99 (0.97, 1.03)
Heterogeneity						
Structured (*u*)		0.331 ± 0.363		0.02		
Unstructured (*v*)		0.483 ± 0.258		0.01		

Spatial Bayesian CAR analyses of locally acquired DF were conducted with and without an LGA that had an extreme value for locally acquired DF cases, and the model estimated without the extreme observation was slightly better fit than the model with the extreme observation (the DIC decreased from 135 to 128). However, average rainfall (RR = 1.05; 95% CI: 1.03, 1.08) and maximum temperature (RR = 1.58; 95% CI: 1.03, 2.33) were still significantly associated with locally acquired DF when the outlier was excluded from the model.

**Figure 3:**
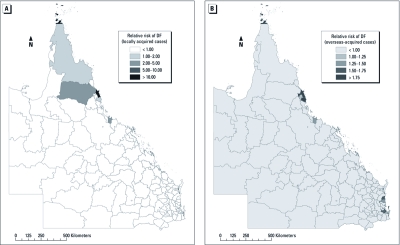
RR of locally acquired DF (*A*) and overseas-acquired DF (*B*) from spatial CAR model.

Posterior estimated RRs of locally acquired and overseas-acquired DF indicated that high-risk areas of locally acquired DF were located in northern Queensland, whereas high-risk areas of overseas-acquired DF were located in coastal cities of Queensland (Figure 3).

**Figure 4:**
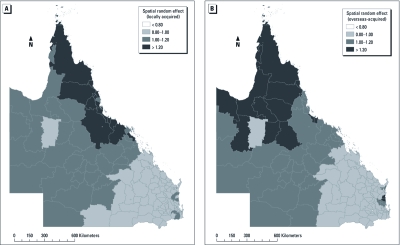
Spatial random effects for incidence of locally acquired DF (*A*) and overseas-acquired DF (*B*).

Estimated residual variation after taking into account the socioecological variables indicated that high-incidence LGA clusters for locally acquired DF were located in northern Queensland, whereas high-incidence LGA clusters for overseas-acquired DF were located in northern and southeastern Queensland (Figure 4). Figure 3 is based on the raw data and shows the overall spatial variability within the dataset. Figure 4 shows the spatial random effect, which, as described above, is the component of variation that is spatially structured (i.e., demonstrates spatial autocorrelation), after accounting for the model covariates. It is, in effect, a spatially smoothed representation of the residual risk after accounting for the covariates.

## Discussion

The results of this study indicate that socioecological factors may have played a significant role in the transmission of DF in Queensland, Australia. DF risk is spatially clustered with different patterns for locally acquired and overseas-acquired cases. There appeared to be different socioecological drivers of locally acquired and overseas-acquired DF. Clusters for locally acquired DF indicated high-risk areas in northern Queensland, whereas clusters for overseas-acquired DF indicated high-risk areas in northern and southeastern Queensland. Therefore, identifying locally acquired and overseas-acquired DF is crucial for developing an integrated early warning system for DF.

Social and economic factors may affect DF transmission directly or indirectly. Tourism and travel have become important mechanisms for facilitating the spread of DF and its vectors (Wilder-Smith and Schwartz 2005). In this study, we found that the average score of SEIFA in an LGA was associated with overseas-acquired DF in Queensland. The results suggest that a higher average SEIFA score (indicating LGAs with higher average socioeconomic status) is associated with an increase in the number of overseas-acquired DF cases. People in the higher socioeconomic groups may be more likely to engage in recreational activities such as camping and overseas travel that may increase the risk of becoming infected with DF. There was little evidence of a relationship between rainfall and DF for overseas-acquired cases, which is consistent with expectations given that local environmental conditions would not be expected to influence the risk of becoming infected while overseas. Maps of the spatially structured random effect indicate residual spatial clustering that is not explained by the socioecological factors included in the models. Bayesian CAR methods can incorporate spatial correlation and uncertainty into the modeling process by including unknown parameters as random variables (Zacarias and Andersson 2010). This approach compensates for residual variability resulting from spatial variation in parameters that were not included in the models, such as land use, urbanization, air-conditioning use, population density, and water storage practices (Hu et al. 2010c).

Temperature and rainfall were associated with the incidence of locally acquired DF in Queensland. Rainfall has also been identified as a contributing factor in the transmission of DF (Banu et al. 2011; Hurtado-Diaz et al. 2007). All mosquitoes including *Ae. aegypti* have aquatic larval and pupal stages and therefore require water for breeding. A few studies have suggested that the greatest increase in *Ae. aegypti* density occurs at the onset of a rainy season (Keating 2001; Lu et al. 2009; Nagao et al. 2003). Temperature can also affect pathogen replication, maturation, the period of infectivity, and the vector’s geographic range or distribution. Higher temperatures accelerate the rate of development of the DF arboviruses, thus increasing the proportion of mosquitoes that are infectious (Patz et al. 1998). It has been suggested that global climate change will have an effect on the future spatial and temporal distribution of DF (McMichael et al. 2006). As climate change continues, there are some concerns that the endemic range of DF will expand geographically (Hopp and Foley 2001). Increasing temperatures could increase the transmission potential and prevalence of DF and extend the season during which DF transmission occurs (Patz 2001). The relative importance of environmental versus social variables on the risk of infection is unclear (Russell 2009; Russell et al. 2009). With the incidence rates of DF continuing to increase, the relative importance of and interaction between environmental and climatic factors need to be elucidated. A recent study suggested that the expanded use of large rainwater tanks throughout urban regions of Australia may have a greater impact on vector distributions than on the direct effects of warming in the future (Beebe et al. 2009). Reiter et al. (2003) suggested that the low prevalence of DF is primarily due to economic and behavioral factors (e.g., use of air-conditioning) rather than climatic factors. Another study suggested that effects of global climate change on DF will vary among different local areas (Johansson et al. 2009).

Hales et al. (2002) used logistic regression to model the presence or absence of DF on the basis of 1961–1990 climate reports. They concluded that the geographical limits of DF transmission are strongly determined by climate. The model results were applied to future climate change situations to generate projections of DF in the 2050s and 2080s. Rogers et al. (2006) used nonlinear discriminant analysis to capture the covariance characteristics of sites of DF presence and absence. However, such models do not account for many nonclimate aspects of the future world, and spatial autocorrelation was not completely removed by these studies.

Transmission of DF is determined by many factors, including social, economic, climatic, and ecological conditions and human immunity (McMichael et al. 2006). Climate change might affect the geographic range of various vector-borne diseases, including DF, although empirical evidence needs to be further established. Rainfall has become more variable globally, and the frequency of intense rainfall has increased in some areas. Our previous results suggested that a decrease in the Southern Oscillation Index, which is a standardized index based on the observed sea level pressure differences between Tahiti and Darwin, Australia (i.e., warmer and drier conditions), was significantly associated with an increase in the monthly numbers of postcode areas with reported DF cases in Queensland, Australia (Hu et al. 2010a). Therefore, a dynamic and integrated early warning system based on climate might help risk managers and local public health authorities identify the communities at increased risk of DF. The advantage of this approach would be to plan DF control and prevention programs well in advance rather than waiting for the occurrence of outbreaks during epidemic seasons.

The DIC was employed for model comparison in this study. Other measures can also be used for model comparison, such as the logarithmic score (LS) and its variants (the cross-validated LS, continuous ranked probability score, score regression, and mean Brier score) (Gneiting and Raftery 2007; Roos and Held 2011). These measures focus primarily on the log likelihood and predictive fit, whereas the DIC is an approximation of the Bayes factor that is an accepted measure for Bayesian model evaluation, particularly for models without many random effects. However, in practice, for models such as the one considered here, there is little difference in model choice based on the DIC and LS (Riebler et al. 2011).

The strengths of this study are that, first, a sophisticated Bayesian spatial model was used to evaluate the difference in the potential predictors between locally acquired and overseas-acquired cases of DF in Queensland. Second, comprehensive and detailed information on socioecological factors by LGA was linked across the whole state and incorporated into the statistical models. Finally, the results from this study may have important implications for public health decision making in identifying risk factors and high-risk areas to control and prevent DF infection.

This study has two major limitations. First, measurement and information biases are possible in this type of ecological study. For example, underreporting would have been likely if people infected by DF had subclinical conditions and did not seek medical attention. Second, little biological or behavioral information was available on community- or individual-level factors (e.g., mosquito population densities, human behaviors, and population immunity) that may potentially confound associations between the socioecological characteristics examined and DF transmission. For example, we could not account for differences in the use of air-conditioning. If the global warming trend continues, air-conditioning may become even more prevalent, which may decrease the probability of DF transmission by decreasing time spent outdoors or exposure to vectors that enter homes through open windows.

The overall findings of this study support the notion of different socioecological drivers of locally or overseas acquired DF. An early warning system for DF based on a Bayesian spatial model would facilitate the early identification of impending epidemics, which could lead to a more rapid response than is possible currently, thereby reducing the magnitude and health and economic impact of epidemics. Novel methods developed in this study may have wide applications in other infectious disease control and risk-management programs, environmental health decision making, and public health practices.
